# Exploiting CRISPR Cas9 in Three-Dimensional Stem Cell Cultures to Model Disease

**DOI:** 10.3389/fbioe.2020.00692

**Published:** 2020-06-24

**Authors:** Sneha Gopal, André Lopes Rodrigues, Jonathan S. Dordick

**Affiliations:** ^1^Department of Chemical and Biological Engineering, Center for Biotechnology & Interdisciplinary Studies, Rensselaer Polytechnic Institute, Troy, NY, United States; ^2^Department of Bioengineering and iBB-Institute for Bioengineering and Biosciences, Instituto Superior Técnico, University of Lisbon, Lisbon, Portugal; ^3^Department of Biomedical Engineering, Rensselaer Polytechnic Institute, Troy, NY, United States; ^4^Department of Biological Sciences, Rensselaer Polytechnic Institute, Troy, NY, United States

**Keywords:** pluripotent stem cell, 3D cell culture microsystem, organoids, microscale 3D printing, patient derived cells (PDCs)

## Abstract

Three-dimensional (3D) cell culture methods have been widely used on a range of cell types, including stem cells to modulate precisely the cellular biophysical and biochemical microenvironment and control various cell signaling cues. As a result, more *in vivo*-like microenvironments are recapitulated, particularly through the formation of multicellular spheroids and organoids, which may yield more valid mechanisms of disease. Recently, genome-engineering tools such as CRISPR Cas9 have expanded the repertoire of techniques to control gene expression, which complements external signaling cues with intracellular control elements. As a result, the combination of CRISPR Cas9 and 3D cell culture methods enhance our understanding of the molecular mechanisms underpinning several disease phenotypes and may lead to developing new therapeutics that may advance more quickly and effectively into clinical candidates. In addition, using CRISPR Cas9 tools to rescue genes brings us one step closer to its use as a gene therapy tool for various degenerative diseases. Herein, we provide an overview of bridging of CRISPR Cas9 genome editing with 3D spheroid and organoid cell culture to better understand disease progression in both patient and non-patient derived cells, and we address potential remaining gaps that must be overcome to gain widespread use.

## Introduction

Stem cells are defined by their long-term self-renewal and their ability to differentiate into specialized progeny (Reya et al., 2001). Pluripotent stem cells (PSCs) can adopt any cellular fate from the three germ layers (ecto-, meso-, and endoderm) (Shamblott et al., 1998). Based on their tissue of origin, stem cells can be classified as embryonic (ESC), which are collected from the inner cell mass of the blastocysts (Kim et al., 2020), or induced pluripotent (iPSC), which are somatic cells reprogrammed toward a more primitive state (Nakagawa et al., 2008). Multipotent stem cells, such as neural stem cells (NSC) and mesenchymal stem cells (MSC), have more limited differentiation capacity but can still generate multiple cell types (Zuk et al., 2002; Donato et al., 2007). Adult stem cells are found in various tissues in the body and serve to replace any tissue lost due to damage or injury (Young and Black, 2004).

The advent of genome engineering tools like Zinc Finger Nucleases (ZFNs), Transcription Activator-Like Effector Nucleases (TALENs) and most recently the Clustered Regularly Interspaced Short Palindromic Repeats Palindromic Repeats (CRISPR) Cas systems have vastly expanded our ability to modify gene expression (Mani et al., 2005; Xiao et al., 2013). In particular, the CRISPR Cas system has revolutionized our ability to perform gene knockout and gene regulation with a high degree of specificity with Cas9 (CRISPR associated protein 9) and dCas9 (deactivated Cas9), respectively (Doudna and Charpentier, 2014). The relative simplicity of this technique has facilitated the study of stem cells in a context dependent manner, particularly using more physiologically relevant 3D cell culture platforms.

Although, early embryonic development has been broadly studied using conventional two-dimensional, cell monolayer platforms (Chambers et al., 2009, 2016; Lian et al., 2012) these systems do not fully recapitulate *in vivo* behavior (Kwon et al., 2014; Duval et al., 2017; Nierode et al., 2019). Conversely, three-dimensional (3D) systems, such as spheroids and organoids, demonstrate a high degree of maturation and functionality. These structures are often generated by the formation of stem cell aggregates known as embryoid bodies (Rungarunlert et al., 2009). This is due to more *in vivo*-like signaling and biophysical gradients, as well as cell–cell contacts, that can impact the many transcriptional networks (Tekin et al., 2018; Branco et al., 2019) and metabolomic pathways inside the cell (Fong et al., 2016; Correia et al., 2018).

Herein, we focus on the application of CRISPR Cas9 tools to knockout/knock-in genes in pluripotent and adult stem cells for disease modeling. A broad overview is provided on the generation of 3D models and a mechanistic understanding of the CRISPR Cas9 technology, and this is followed by the use of gene knockouts to interrogate human diseases. Key to the latter is how CRISPR Cas9 has been used to develop physiologically relevant disease models that affect multiple organ systems. Finally, existing gaps are addressed leading to opportunities for future research.

## Engineering Strategies for 3D Organoid Models for Stem Cells

The current definition of an organoid is a self-organized, 3D multicellular structure that possesses functional attributes to its *in vivo* counterpart (Simian and Bissell, 2017). While organoids have been examined for decades in tumor-derived explants (Smith and Cochrane, 1946), more recently, organoids have been developed to include self-organized structures derived from pluripotent and adult stem cells. This self-organization is intrinsically dependent on biochemical factors like morphogens, small molecules and growth factors that are delivered in a spatiotemporal fashion as well as on biophysical stimuli provided by cell–cell and cell-extracellular matrix (ECM) interactions (extensively reviewed in Brassard and Lutolf, 2019; Silva et al., 2019).

**FIGURE 1:**
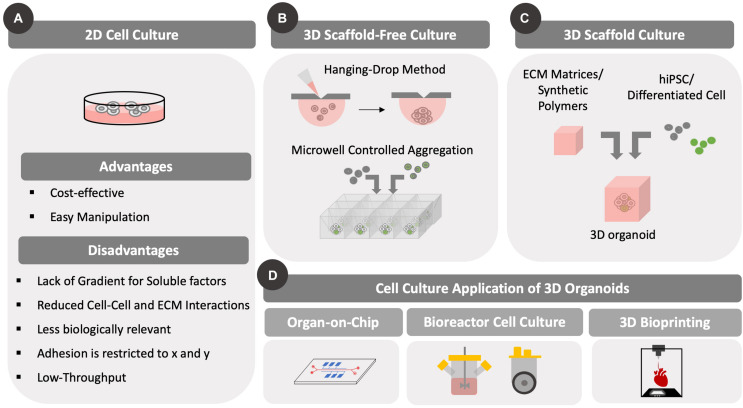
Different types of cell culture formats. Differences between 2D and 3D cell culture methods are highlighted. Importantly, 3D cell culture formats have been developed to accommodate static and/or dynamic (with fluid flow/mixing) designs. The development of 3D culture methods has been prompted by scaffold-based and scaffold-free approaches that can be used for various culture methods, including microfluidic bioreactors and bioprinting. **(A)** Conventional 2D cell culture formats are illustrated along with advantages and disadvantages. Cells grow as a 2D monolayer with cell–cell contacts across a single surface. **(B)** Several scaffold-free approaches are highlighted including hanging drop and controlled aggregation methods that use gravity to assemble cells in 3D. **(C)** Scaffold based approaches include encapsulation of cells in synthetic or natural matrices that provide support to the cells and allow them to remain suspended. **(D)** The two methods of 3D cell culture have been adapted to both perfusion and static cultures in the form of microfluidic organ-on-chip platform/bioreactor platforms or as bioprinting on a surface, respectively.

Several protocols have been established to promote cellular assembly of 3D structures to recapitulate organ level functions *in vitro* with both scaffold-based and scaffold free approaches as shown in Figure 1. In scaffold-based approaches, the microenvironment of naive tissue is provided by matrices that replicate specific *in vivo* physical and biochemical stimuli. Early approaches relied on naturally derived matrices from decellularized tissue (Dye et al., 2015). For example, Sato et al. (2009) used laminin-rich Matrigel as an encapsulating matrix to support epithelial growth of mouse intestinal crypts. An alternative approach involved an air-liquid interface that provides better oxygenation to 3D intestinal cell cultures (Ootani et al., 2009). In this study, a collagen matrix was used to encapsulate primary intestinal cells in the presence of myofibroblast, which provided essential cues to recapitulate an intestinal stem cell niche allowing cell growth and differentiation with the additional external delivery of WNT and Notch signaling molecules. In the context of hPSC, Lancaster et al. (2013) and Lancaster and Knoblich (2014) have developed a widely used approach, in which cerebral organoids were prepared for modeling microcephaly via knockdown RNA interference (iRNA) on hiPSC lines with disease-associated Cyclin Dependent Kinase 5 Regulatory Subunit Associated Protein 2 (CDK5RAP2) mutations. By embedding embryoid bodies in Matrigel following neural commitment, the authors were able to achieve interdependent brain regions following formation of functional cortical neurons (Lancaster et al., 2013). Further in-depth transcriptomic analysis and DNA methylome sequencing demonstrated that these cerebral organoids share a similar expression profile and epigenetic signature with their fetal counterparts, namely similar gene expression patterns for neural progenitor self-renewal, differentiation, ECM production, adhesion and migration, and thus demonstrating how organoids could be used for neurodevelopmental studies (Camp et al., 2015; Luo et al., 2016).

Although Matrigel is widely used in stem cell organoid culture, its heterogenous composition poses a disadvantage to study specific spatial temporal cues that govern cell organization. As an alternative, hydrogels can be used to form 3D polymeric networks that support organoid culture under defined conditions. Lindborg et al. (2016) developed a hyaluronic acid (HA)-based hydrogel to grow cerebral organoids and avoid matrix variability. Hydrogels can also be functionalized with ECM proteins, such as collagen to mimic a defined cell microenvironment (Takezawa et al., 2004; Ootani et al., 2009; Lindborg et al., 2016). In addition, soft-lithography, including microcontact printing, has been used to promote cell aggregation in highly organized 3D structures (Rivron et al., 2012; Berger et al., 2015; Filipponi et al., 2016; Foncy et al., 2018).

Polysaccharides like alginate have also been shown to support growth of hiPSC-derived and patient-specific organoids, rendering similar phenotypic traits comparable to Matrigel-grown organoids (Lu et al., 2017; Wilkinson et al., 2017; Broguiere et al., 2018; Capeling et al., 2019). Osteogenesis and angiogenesis could be achieved in an artificial sphere-shaped organoid using mesenchymal stem cells (MSCs) and human umbilical vain endothelial cells (HUVECs) (Zhao et al., 2018). Such an approach may provide a functional delivery method of osteogenic factors for bone injury, which could be personalized by using patient-specific MSC or hiPSC. Other studies using alginate-based matrices include hiPSC-derived organoids such as to mimic a vascularized-myocardium on-chip (Zhang et al., 2016) as well as a 3D patient-specific lung organoid to study idiopathic pulmonary fibrosis (Wilkinson et al., 2017).

The same principle of tethering ECM components to biopolymers can also be applied to synthetic hydrogels like polyethylene glycol (PEG). Gjorevski et al. (2016) compared different designs of PEG-based defined matrices for intestinal stem cell organoid culture. PEGs were functionalized with HA, fibronectin, collagen or RGD peptide domain responsible for cellular adhesion to the ECM matrix, and examined resulting organoids *in vitro* (Gjorevski et al., 2016). Interestingly, there have been studies that do not include ECM components in matrix design. Cruz-Acuña et al. (2017) developed a four-armed PEG-macromer that was used to generate hPSC-dervied lung organoids with *in vivo*-like epithelial morphology. Likewise, Candiello et al. (2018) demonstrated that only PEG-crosslinked amikacin hydrogel was sufficient to induce a 3D self-organization of hESC-derived pancreatic cells, with further phenotypic maturation achieved upon addition HUVECs that promoted endocrine-like function.

Although the aforementioned studies do not introduce ECM components in matrix design, it should be noted that such components are still produced within the organoid. Indeed, this is the premise for scaffold-free methods. In such cases, cells can be cultured in small droplets of media (hanging drop method) and forced to aggregate based solely on gravitational force (Hsiao et al., 2012). This approach has been used widely to establish 3D models for cardiac diseases (Fennema et al., 2013; Beauchamp et al., 2015) and cancer (Amaral et al., 2017; Eder and Eder, 2017). Additional scaffold-free approaches include the formation of free-floating aggregates in exclusively non-adherent culture conditions (Eiraku et al., 2008; Paşca et al., 2015; Birey et al., 2017; Yoon et al., 2019), as well as the controlled aggregation of cells using microwells fabricated through soft-lithography techniques (Ungrin et al., 2008; Antonchuk, 2013; Dahlmann et al., 2013; Mitsunaga et al., 2017). Microfluidic devices have been employed to achieve physiologically relevant 3D organoid models. Herein, different groups have established protocols to study neural tube formation *in vitro* (Demers et al., 2016), perform drug screening using hepatic organoids (Novik et al., 2010; Au et al., 2014; Ortega-Prieto et al., 2018) and to develop 3D cardiac models (Shin et al., 2016; Devarasetty et al., 2017).

## Crispr Cas9 Systems

The CRISPR Cas9 system is made up of two components - a Cas9 nuclease and a short guide RNA (sgRNA) (Cho et al., 2013). Discovered as a part of the bacterial adaptive immune system, CRISPR Cas9 has been used widely to both knock out and regulate gene expression in now many cell types (Maeder et al., 2013; Qi et al., 2013; Ran et al., 2013b). A CRISPR knockout can be accomplished by designing a 20-nucleotide (nt) sgRNA sequence that can bind to a specific gene of interest through complementary base pairing (Ran et al., 2013b; Shalem et al., 2014). Upon successful target identification, the Cas9 nuclease induces a double strand break (DSB) in the presence of a three-nucleotide protospacer adjacent motif (PAM).

The DSB causes the cells to repair the break by inserting and deleting random sequences at the site of the break through an error prone repair mechanism called Non-Homologous End Joining (NHEJ) (Shan et al., 2014). This process is often exploited when the CRISPR Cas9 system is used in stem cells to create different disease-specific models (Lin et al., 2018; Latour et al., 2019; Strikoudis et al., 2019). In addition, through homology dependent repair (HDR) a specific gene or sequence can be inserted at the site of the break if that sequence is simultaneously introduced to the cell with the CRISPR Cas9 system (Zhang et al., 2017). While this process occurs less frequently than NHEJ, HDR has been leveraged to generate multiple distinct reporter cell lines with applications in screening and cell modeling (Maruyama et al., 2015; Sluch et al., 2015; Wu et al., 2016; Zhou et al., 2016; Zhang et al., 2017). Two genomic editing tools were commonly used before CRISPR Cas9 - Zinc Finger Nucleases (ZFN) and Transcription Activator Like Effector Nucleases (Hai et al., 2014). These tools use complex nuclease design methods depending on the region to be targeted or they employ difficult cloning tools. Conversely, CRISPR Cas9 requires the design of just a 20-nt RNA sequence to ensure targeted genome editing (Hruscha et al., 2013). In addition, Cas9-mediated editing can introduce mutations at specific sites (Hwang et al., 2013; Ran et al., 2013a; Chiang et al., 2016).

Gene regulation with CRISPR Cas9 can be accomplished by using CRISPR interference (CRISPRi) and CRISPR activation (CRISPRa) (Charpentier and Marraffini, 2014; Du and Qi, 2016; Kampmann, 2017). In CRISPRi/a, several point mutations in the nuclease domain create a catalytically dead dCas9 (Larson et al., 2013; Guilinger et al., 2014). The dCas9 is still able complex with the sgRNA but no longer has any cleavage activity. The complex, therefore, binds to the DNA target without causing a DSB it at the specific site (Qi et al., 2013). In the case of CRISPRi, the sgRNA-dCas9 complex blocks the action of RNA polymerase and prevents transcription elongation (Mandegar et al., 2016). The efficiency of repression can be improved by further modification of the dCas9 to include a repressive domain, such as the Kruppel-associated box (KRAB) (Gilbert et al., 2013; Kearns et al., 2014; Genga et al., 2016). For gene upregulation, dCas9 can be fused to activator domains and targeted to promoter/enhancer regions of genes (Dominguez et al., 2015). Several systems have been generated, including direct dCas9 fusions (e.g., VPR, a tripartite fusion of VP64, p65, and Rta) (Chavez et al., 2015), utilizing a protein scaffold (e.g., SunTag system) (Papikian et al., 2019), and incorporating an RNA scaffold [e.g., Synergistic Activation Mediator complex (SAM)] (Konermann et al., 2015).

**FIGURE 2:**
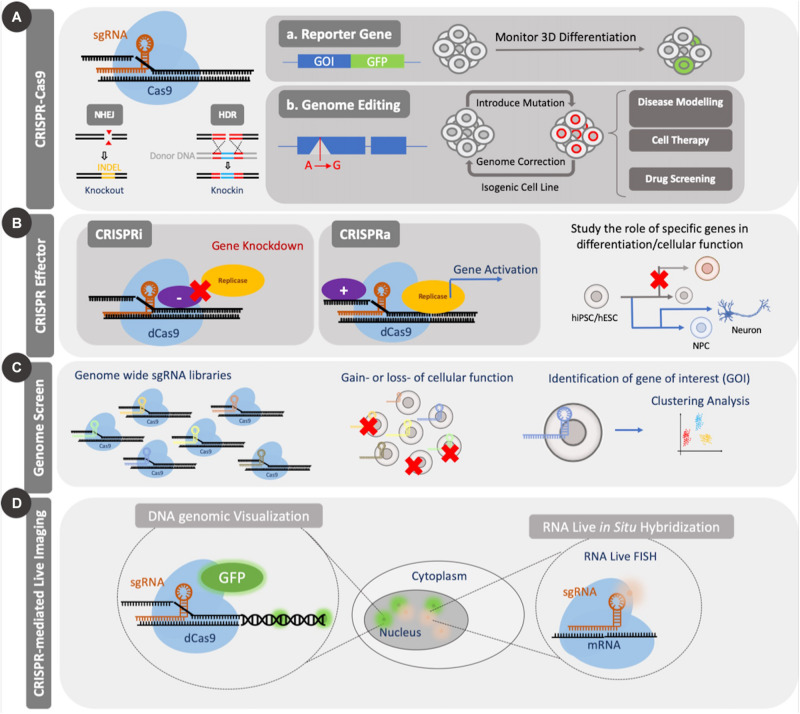
Applications of CRISPR Cas9. CRISPR Cas9 can be used to introduce genetic mutations by causing a double strand break (DSB). The nuclease-null version, dCas9, can be coupled to effector domains to activate or repress gene expression or can be coupled to fluorescent proteins for imaging of genomic elements. CRISPR Cas9 knockout and effector tools have been used for screening applications. **(A)** Standard CRISPR Cas9 genome editing through introduction of mutations can be used to study the role of several genes during differentiation or to create disease models for enhanced mechanistic understanding or drug screening. **(B)** Application of dCas9 to genomic regulation in the form of CRISPRi/a can be used to study both differentiation and cellular function. **(C)** Large scale genomic screens using CRISPR Cas9/dCas9 can be used to identify essential genes for several cellular processes through gain or loss of function studies. Such screens enable identification of candidate genes from a pool for further investigation. **(D)** Coupling dCas9 to fluorescent domains, like GFP, enable facile visualization of specific regions of the genome and temporal tracking.

As a result of the growing success of genome regulation with CRISPRi and CRISPRa, the nuclease null version of Cas9 has been used in other applications including single nucleotide editing, chromatin modifications, imaging genomic sequences, evaluating regulatory sequences, among others (Chen et al., 2013; Anton et al., 2014; Thakore et al., 2015; Gaudelli et al., 2017; Takei et al., 2017) (Figure 2). The earliest example of single base editing was carried out with dCas9 coupled to an apolipoprotein B mRNA editing enzyme, a catalytic peptide where a cytosine group was converted to uracil or thymine (Komor et al., 2016). Other base editors that can carry out adenine–thymine base pair changes to cytosine-guanine base pairs have also been developed (Gaudelli et al., 2017). Such tools are useful from a therapeutic standpoint as they have the ability to be used to correct diseases that arise from single base pair changes in a gene therapy strategy. For a more thorough assessment of the different applications of dCas9-mediated control of cells, readers are referred to the review by Pickar-Oliver and Gersbach (2019).

In addition to these varied applications, CRISPR Cas9 has been adapted to genome wide screens like those carried out with RNA interference (RNAi) (Mohr and Perrimon, 2012; So et al., 2019). As a result, they have been adapted to identify genes involved in conferring cell viability, drug resistance and promoting differentiation or self-renewal in stem cells (Shalem et al., 2015; Ihry et al., 2019). Such screens are typically performed in a pooled or an array-based format (Tian et al., 2019). The former method delivers all of the sgRNAs simultaneously and requires a selection post-introduction to isolate the cells that take up a specific sgRNA, while the latter delivers one sgRNA to one group of cells, thereby removing the need for selection (So et al., 2019). While there have been several reports that have employed genome wide pooled screens in stem cells in 2D formats, more recent studies have used these tools to study 3D organoids that encapsulate adult stem cells (Michels et al., 2020; Ringel et al., 2020).

There are three ways to incorporate CRISPR Cas9 to study stem cells in 3D organoids. One method relies on genome modification of cells prior to their encapsulation in a matrix (Latour et al., 2019), while a second method involves introduction of a Cas9-sgRNA complex in organoids dissociated into single cells during infection and subsequent reformation of 3D structures (O’Rourke et al., 2017). The third method involves direction delivery of Cas9 and sgRNA to the organoids without generating single cells (Matano et al., 2015). Of the three methods, the first two have been employed more widely due to difficulties associated with efficient gene delivery through 3D matrices (Laperrousaz et al., 2018). Since hydrogel matrices can often pose diffusional limitations to highly charged, relatively large non-viral gene delivery vectors, limitations remain in using CRISPR Cas9 to study organoids. Nevertheless, viral vectors are being employed together with nucleofection and electroporation to deliver genes to spheroid and organoid cultures (Matano et al., 2015; Bian et al., 2018; Garita-Hernandez et al., 2020). The development of novel gene delivery tools can expand our ability to carry out direct genomic editing of organoids.

## Development of Disease Models Using Crispr Cas9 Knockout

**FIGURE 3:**
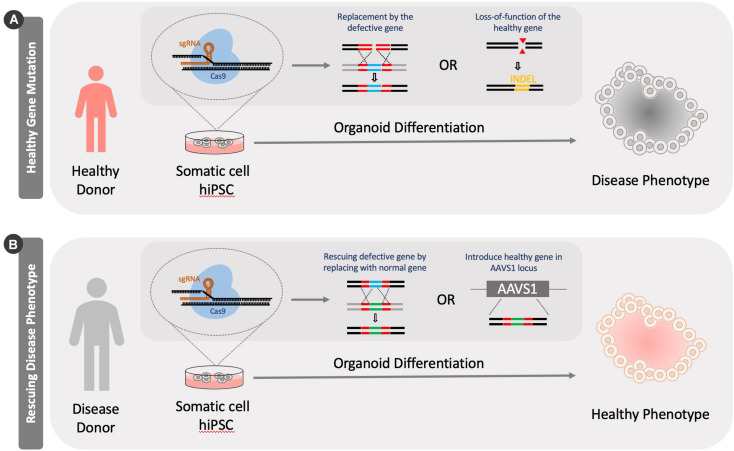
Generation of disease models using CRISPR Cas9 knockout tools. Models can be generated by either knocking out a healthy version of a gene in cells or by rescuing a defective gene. **(A)** A healthy person’s somatic cells are reprogrammed into a pluripotent stage. Specific disease causing mutations can be introduced to genes in these cells by either knocking in a defective genes or introducing INDELS through genome editing. **(B)** Alternatively, a diseased patient’s cells can be isolated and reprogrammed into a pluripotent stage. These cells can be then fixed by correcting the mutation by knocking in a functional version of the gene or introducing healthy version at the adeno-associated virus integration site (AAVS1).

The use of hiPSCs/hESCs for the development of human disease models is built on their ability to adopt essentially any cellular fate through differentiation. Therefore, hiPSCs/hESCs may serve as an unlimited source of disease relevant cells. These cells can then be used to better understand pathologies associated with specific diseases, as well as to search for potential treatment options. Several diseases including Parkinson’s disease, amyotrophic lateral sclerosis (ALS), cystic fibrosis, cardiac fibrosis, polycystic kidney disease, among others, have been studied using hiPSCs/hESCs and other cell types (Hofherr and Köttgen, 2013; Fujimori et al., 2018; Pollard and Pollard, 2018; Lee et al., 2019; Stoddard-Bennett and Reijo, 2019). Genome editing using CRISPR-Cas9 in 3D organoids has been used for such disease modeling, either by modifying a parental undifferentiated stem cell line or by rescuing a gene that is involved in causing the disease phenotype (Figure 3).

### Generating Cells With Disease-Carrying Mutations

To introduce disease mutations and potentially mimic disease phenotypes, CRISPR Cas9 has been used to knock out various genes in healthy stem cells. Several types of organoids, including those consisting of brain (Kim et al., 2019), lung (Strikoudis et al., 2019), kidney (Freedman et al., 2015), intestinal (Jung et al., 2019), and other stem cells have been generated from these knockout stem cell types to better model *in vivo* environments. A majority of studies have focused on better understanding brain disorders with cerebral organoids and hence this is a major focus of this section.

A major advantage of genome engineering is the ability to study diseases in experimentally relevant human cell types. For example, patient-specific iPSCs were used to create a disease model of frontotemporal dementia (FTD), wherein CRISPR Cas9 was used to introduce mutations in the MAPT gene (Nakamura et al., 2019). There were several changes noted in generated neurons due to the mutation including lower phosphorylation levels in the tau protein, which resulted in structural reorganization of the microtubules as well as reduced numbers of mitochondria in the cells.

In addition, the specificity of gene targeting with CRISPR/Cas9 enabled the generation of similar mutations that are found in patients carrying a specific disease. This was shown with a cerebral organoid disease model for GM1 ganglioside storage disease that results in extensive buildup of a glycosphingolipid ganglioside due to a non-functional cellular β-galactosidase (Tonin et al., 2019). Human iPSCs were used to prepare isogenic cell lines with the disease carrying mutations in specific exons where most mutations for the disease were recorded in patients (Latour et al., 2019). Following gene editing, increased levels of GM1 were observed in mutated organoids over non-mutated controls leading to GM1 ganglioside storage disease phenotypes recapitulated in gene-edited organoids.

Phenotypes of major neurodegenerative diseases like Alzheimer’s disease and Parkinson’s disease can be recapitulated in a 3D organoid environment by CRISPR Cas9-mediated editing (Lin et al., 2018; Kim et al., 2019). For Alzheimer’s disease, cerebral organoids were prepared from hiPSCs that harbored the disease causing variant of apolipoprotein E (APOE4), which was found to show ∼2 fold increase in the accumulation of amyloid β than control organoids that had the APOE3 gene variant (Lin et al., 2018). For Parkinson’s disease, Kim et al. (2019) generated a single point base mutation to model a specific form of the disease, the LRRK2-associated sporadic disease. Cerebral organoids generated from mutated hiPSCs were then found to yield several different phenotypes that were observed with Parkinson’s disease and also used to identify a disease relevant gene encoding for the thioredoxin interacting protein (TXNIP) (Kim et al., 2019). They further showed that the gene edited dopaminergic neurons showed enhanced cytotoxicity as indicated by a ∼20% increase in caspase-3 activity. These studies are especially relevant since CRISPR-edited iPSCs have the ability to generate a near limitless supply of cells for studying these diseases as opposed to patient derived adult cells, which are far more finite.

Structural conditions like macrocephaly, microcephaly and cell migration can be studied more accurately with 3D organoid models incorporating CRISPR induced mutations than with animal models. To this end, Li et al. (2017) generated a Phosphatase and Tensin Homolog (PTEN) gene knockout model to activate PTEN-AKT and cause 3D organoids to display a macrocephaly phenotype. The authors were thus able to address several interspecies differences in this pathway including stark differences in the expansion and folding mechanism of human organoids when compared to mice. Therefore, CRISPR Cas9 can be used to address some of the issues associated with translating results between multiple species by enabling study in the more relevant human cell lines.

Finally, genome modification to multipotent stem cell types, including neural stem cells (NSCs), have been used to model diseases like cancer in cerebral organoids (Bian et al., 2018). Direct introduction of CRISPR Cas9 for mutating several tumor suppressor genes in 3D embryoid bodies was performed at the NSC stage to identify key genes, which when mutated, resulted in abnormal growth associated with various cancers. Mutations in certain genes resulted in rapid proliferation of the organoids in culture. Further investigation revealed mRNA changes in the expression of several genes between mutated cancerous organoids and non-cancerous organoids. Such disease models have yielded a wealth of information on how cells change with tumorigenesis and how this information could be leveraged to generate better potential drug candidates to reduce tumorigenesis (Luo, 2016).

A cohort study conducted by van Rijn et al. (2018) is an interesting example of how CRISPR-engineered organoids are useful in determining genotypic-phenotypic associations. This study provided insights of the mechanism by which mutations in the diacylglycerol-acyltransferase 1 (DGAT1) gene is associated to congenital intestinal disorders. The authors used CRISPR-Cas9 to generate knockouts of the DGAT1 gene on cells obtained from duodenal biopsies of three healthy individuals and compared lipid production to patient-derived organoids that contain the mutations leading to similar results.

Kidney organoids have been studied in detail using CRISPR Cas9-edited hiPSCs to model renal diseases. A knockout of phosphoinositide dependent kinase 1 (PDK1) and phosphoinositide dependent kinase 2 (PDK2) used to model disease pathology of polycystic kidney disease showed enhanced cyst formation when cells were grown into kidney organoids (Freedman et al., 2015). Some of the lesser-studied organoid models with CRISPR Cas9 include lung organoid models where only one group has done major work to study a lung disease in 3D format using CRISPR Cas9 (Strikoudis et al., 2019). Nevertheless, this shows that Cas9 mediated knockout can be used to study multiple different organoid systems. A list of studies that have utilized CRISPR Cas9 to introduce disease causing mutations in stem cells for further study in 3D cell cultures are given in Table 1.

**TABLE 1:** Examples of CRISPR Cas9 to introduce disease causing mutations.

**Edited cell type**	**Type of organoid**	**Gene knockout**	**Disease modeled**	**Patient derived (Y/N)**	**References**
iPSC	Cerebral	MAPT	Frontotemporal dementia	Y	Nakamura et al., 2019
iPSC	Cerebral	GLB1	GM1 ganglioside storage disease	N	Latour et al., 2019
iPSC	Cerebral	RS1	X-linked juvenile retinoschisis	Y	Huang et al., 2019
iPSC	Cerebral	AXL	Zika virus	N	Wells et al., 2016
ESC	Cerebral	LIS1	Lissencephaly	N	Karzbrun et al., 2018
iPSC	Cerebral	APOE3	Alzheimer’s disease	N	Lin et al., 2018
iPSC	Cerebral	LRRK2	Parkinson’s disease	N	Kim et al., 2019
ESC	Cerebral	PTEN	Zika virus	N	Li et al., 2017
NSC derived from ESC	Cerebral	Multiple tumor suppressor genes	Brain tumor	N	Bian et al., 2018
iPSC	Cerebral	TSC1, TSC2	Tuberous sclerosis	N	Blair et al., 2018
Organoids from hESC	Cerebral	TP53	Glioblastoma	N	Ogawa et al., 2018
iPSC	Cerebral	DCHS1, FAT4	Neuronal heterotopia	Y	Klaus et al., 2019
iPSC	Cerebral	CLN3	Neuronal ceroid lipofuscinosis	N	Gomez-Giro et al., 2019
iPSC	Cerebral	CDH8	Schizoaffective disorder	Y	Wang et al., 2017
iPSC/ESC	Kidney	PODXL	Podocyte development	N	Freedman et al., 2015; Kim et al., 2017
ESC	Kidney	PKD1, PKD2	Polycystic kidney disease	N	Freedman et al., 2015
ESC	Intestinal	STAT3	Intestinal organoid maturation	N	Jung et al., 2019
ISC	Intestinal	APC, TP53, KRAS, SMAD4	Colorectal cancer	Y	Drost et al., 2015; Matano et al., 2015; O’Rourke et al., 2017; Roper et al., 2017
ISC	Intestinal	MLH1, NTLH1	Colorectal cancer	Y	Drost et al., 2017
ESC	Lung	HPS2, HPS4, HPS8	Hermansky-Pudlak syndrome interstitial pneumonia	N	Strikoudis et al., 2019
ISC	Intestinal	DGAT1	Congenital diarrheal disorder	Y	van Rijn et al., 2018

Genome wide pooled screening tools have only recently been employed to study stem cells in 3D organoid and spheroid cultures (Michels et al., 2020; Ringel et al., 2020). Michels et al. (2020) and Ringel et al. (2020) used adult intestinal stem cells and cancer stem cells to identify genes that prevent cells from responding to transforming growth factor-β (TGF-β) signaling. As a result, pooled CRISPR screens can effectively generate large amounts of data on the roles of several genes simultaneously. These experiments show the possibility of conducting screening experiments with stem cells and can now be expanded for use in studying pluripotent and multipotent stem cells. While a few experiments have been performed in 2D, to our knowledge there are no reported examples in 3D.

Animal models have been useful in helping us study disease progression as well to evaluate treatment options. Xenotransplantation of CRISPR engineered organoids are particularly useful as they do not require complex methods needed to generate mice carrying specific mutations (Roper et al., 2017). Modified tissue xenografts have been studied extensively with human and rodent adult intestinal stem cells (Drost et al., 2015; Matano et al., 2015; O’Rourke et al., 2017; Roper et al., 2017). These genome-edited organoids were used to evaluate tumor progression during cancer development (Drost et al., 2015; O’Rourke et al., 2017). In addition, such studies enable the development of gene expression profiles in tumors and essential mutations needed for cancer metastases (Matano et al., 2015; Roper et al., 2017).

### Generating Healthy Cells With Gene Rescue

CRISPR Cas9 has been used to perform gene rescue and better understand disease pathologies. One of the earliest examples of using CRISPR Cas9 to repair a mutant gene was done using cystic fibrosis transmembrane conductor receptor (CFTR) (Schwank et al., 2013). Adult intestinal stem cells were transfected with Cas9, sgRNA, and wild type CFTR genes to allow for homologous recombination of the corrected gene following which a forskolin based swelling assay was carried out (Schwank et al., 2013). When these cells were grown into intestinal organoids, the corrected organoids displayed increased swelling in comparison to the diseased organoids (non-mutant) when they were subjected to a forskolin based assay. Forskolin swelling assay is commonly used to study cystic fibrosis since diseased cells carry a non-functional CFTR channel that prevents swelling of cells upon exposure to forskolin since the channel is unable to transport chloride ions into the cells (Boj et al., 2017).

Disease correction has also been carried out with CRISPR Cas9 in hiPSCs. This has important implications in regenerative medicine, as such genome editing tools may be used therapeutically. Diseased or mutant gene alleles can be modified into “healthy” versions in reprogrammed hiPSCs, which subsequently can be differentiated to any target cell type that can serve as a replacement to the diseased tissue. Some examples are summarized in Table 2. Gene rescue in hiPSCs has been used to generate multiple organoid types including cerebral (Allende et al., 2018), intestinal (Geurts et al., 2020), kidney (Forbes et al., 2018), and retinal (Deng et al., 2018) organoids.

**TABLE 2:** CRISPR Cas9 examples to rescue genes.

**Cell type**	**Organoid**	**Gene rescued**	**Disease studied**	**Patient derived (Y/N)**	**References**
ISC	Intestinal	CFTR	Cystic fibrosis	Y	Schwank et al., 2013
iPSC	Intestinal	DKC1	Dyskeratosis congenita	Y	Woo et al., 2016
ISC	Intestinal	CFTR	Cystic fibrosis	Y	Geurts et al., 2020
iPSC	Cerebral	HEXB	Sandhoff disease	Y	Allende et al., 2018
iPSC	Cerebral	CNTNAP2	Autism spectrum disorder	N	de Jong et al., 2019
iPSC	Kidney	IFT140	Nephronophthisis related ciliopathy	Y	Forbes et al., 2018
iPSC	Retinal	RPGR	Retinitis pigmentosa	Y	Deng et al., 2018
iPSC	Cerebral	LIS1, YWHAE (14.3.3e)	Miller-Dieker syndrome	N	Iefremova et al., 2017
Hepatocytes from fetal human livers	Hepatic	TP53	N/A	N	Artegiani et al., 2020

In another example of gene rescue, a dyskeratosis congenital (DC) intestinal organoid model was used to identify the Wnt signaling pathway as a potential therapeutic target for the disease (Woo et al., 2016). CRISPR Cas9 corrected DC cells that were isolated from patients showed higher expression of several genes regulated by Wnt signaling. Specifically, Ascl2, a known regulator of intestinal stem cell development (Schuijers et al., 2015) showed differential expression between diseased and corrected organoids (Woo et al., 2016). The success in using intestinal organoids to model diseases has also resulted in newer CRISPR technologies including adenine base editing used to correct disease causing mutations with a large set of patient derived cells (Geurts et al., 2020). Four types of patient derived cells were corrected using a Cas9 base pair editor that allows for switching an adenine–thymine (A–T) base pair to cytidine–guanine base pair (C–G). This work is especially important since a large number disease causing mutations are due to a base pair substitution event (C–G base pair change to A–T) (Gaudelli et al., 2017). Furthermore, the ability to utilize these base-editing tools with multiple different patient samples shows the versatility of CRISPR Cas9 for therapeutic purposes.

Normal cell function was restored in several other organoid models including cerebral (Allende et al., 2018), kidney (Forbes et al., 2018), and retinal (Deng et al., 2018) organoids using CRISPR Cas9. For example, CRISPR Cas9 was used to correct a mutation and rescue a gene involved in causing Sandhoff disease (a lysosomal storage disorder) from patient derived cells (Allende et al., 2018). Specifically, gene edited organoids showed reduced proliferation and did not show developmental abnormalities that were found with the diseased organoids. With kidney organoids, the intraflagellar transport 140 (IFT140) gene was rescued in cells derived from a patient who had nephronophthisis (NPHP)-related ciliopathy (NPHP-RC) (Forbes et al., 2018). There were several structural differences as well as gene expression differences in the organoids, especially in genes involved in the creation of apicobasal polarity, the formation of cell–cell junctions and in the assembly of dynein motor proteins. Finally, several phenotypic changes associated with retinitis pigmentosa were corrected with the use of CRISPR Cas9 mediated editing of retinitis pigmentosa 3 GTPase regulator (RPGR) in 3D retinal organoid model (Deng et al., 2018). The authors were able to reverse several disease-related symptoms including loss of photoreceptors and ciliopathy in the patient derived cells by correcting a frameshift mutation in the RPGR gene.

Alternatively, the safe harbor AAVS1 locus of patient-derived hiPSC cells can be used as a site to introduce the Cas9 encoding gene and a functional version of the gene to be rescued (Oceguera-Yanez et al., 2016; Iefremova et al., 2017). For example, Iefremova et al. (2017) transfected Miller-Dieker syndrome patient-derived hiPSCs with plasmids encoding Cas9 and two other proteins (LIS1 and 14.3.3e) to partially rescue the lissencephaly phenotype. They further showed that the neuroepithelial loops that were generated upon differentiation showed increased loop diameter and apical membrane length in organoids (∼50 μm increase ∼200 μm increase, respectively, for LIS1 rescue and ∼75 and ∼100 μm increase, respectively, for 14.3.3e rescue).

Most of the aforementioned studies described in this section resort to HDR to introduce the gene mutation at a specific site, as this approach relies on homology arms present in the donor plasmid. Nevertheless, NHEJ-mediated mechanisms potentially could be used to generate knock-in functional genes (Artegiani et al., 2020). As a proof-of-concept, Artegiani et al. (2020) developed a CRISPR-Cas9-mediated homology-independent organoid transgenesis method, coined CRISPR-HOT, to mediate precise gene insertion in different types of organoids, namely liver-, and intestinal-derived tissues. When compared to HDR, a 10-fold higher efficiency was achieved with precise in-frame insertions of a gene encoding a fluorescent protein used for C-terminal tagging. Although this approach is a useful tool for reporter cell line generation and labeling cellular structures, it can also be multiplexed with gene knock-out studies, as demonstrated by the loss of TP53 gene in hepatic organoids, resulting in an aberrant mitotic spindle behavior and disorganization of microtubules. Thus, CRISPR-HOT may be useful in generating myriad disease models (Artegiani et al., 2020).

## Conclusion and Future Perspectives

Although remarkable advancement in 3D model development has been achieved, there remain important questions to be answered. Recently, several vascularization processes have been incorporated into 3D models (Kang et al., 2009; Ovsianikov et al., 2011; Kook et al., 2018; Wörsdörfer et al., 2019). Two such methods include bioprinting encapsulated cells in synthetic hydrogels resulting in perfusable networks through two photon polymerization (Ovsianikov et al., 2011) or co-culturing organoids with endothelial cells (Kang et al., 2009; Kook et al., 2018). For a detailed summary of methods used to generate vascular tissue, readers are referred to the review by Grebenyuk and Ranga (2019). Despite progress made toward incorporating vascular structures in 3D organoid models, there are few studies on the use of such tools in understanding disease progression with CRISPR-modified organoids. Combining vascularization with specific disease-causing mutations in 3D may help yield more personalized patient models.

In addition, while the majority of this review has focused on using CRISPR Cas9 knockout tools for studying diseases, some groups have also started using CRISPRi/a for similar studies (Heman-Ackah et al., 2016). This is especially important because CRISPRi/a allows for temporary modifications to gene expression through inducible promoters as opposed to the more permanent gene knockout experiments. Such experiments can rapidly simplify the process of studying diseases in 3D organoids by removing the need for creating multiple different cell lines with and without mutations and also allows for dynamic control of disease phenotypes with a single population of cells. However, most such studies have been carried out in 2D cell formats. As an example, Heman-Ackah et al. (2017) demonstrated that the VPR (a tripartite fusion of VP64, p65, and Rta) domain coupled to dCas9 could be used to induce activation of endogenously expressed SCNA gene in healthy neurons, which led to an eightfold increase of the alpha-synuclein protein associated with Parkinson’s disease (Heman-Ackah et al., 2017). In parallel, the KRAB domain was used to reduce the transcriptional expression of the same protein in diseased-phenotypic neurons that led to a 40% reduction relative to a non-edited Parkinsonian hiPSC-derived neuron (Heman-Ackah et al., 2016). Despite significant improvements in understanding the role of the SCNA gene, it is unclear how it would influence the 3D structure of the organoid, or how the specific microenvironmental cues could potentially influence epigenomic regulation of the edited gene (Lo and Qi, 2017). Modulating gene expression of SCNA or any other gene of interest in organoids and spheroids with CRISPRi/a tools can help address several of these unknowns.

Another important aspect that requires further study in this area is the use of artificial intelligence and machine learning tools coupled to CRISPR-engineered organoids. For instance, groundbreaking work developed by Libby et al. (2019) demonstrate that machine learning algorithms could empirically determine and control the spatial self-organization of multicellular patterns without the need for micropatterning technique or other extrinsic patterning methods. Other studies demonstrated that trained machined learning models could be used to identify possible mutations (indels) generated by CRISPR genome editing and repair (Allen et al., 2018). This could be further used to improve disease models (Shen et al., 2019).

Finally, novel CRISPR Cas tools that utilize other Cas proteins including Cas12a and Cas13 and their catalytically dead counterparts, have been discovered specifically for nucleic acid detection and protein detection (Gootenberg et al., 2018; Bai et al., 2019; Dai et al., 2019). Of these two, Cas13 has the ability to target RNA sequences (Granados-Riveron and Aquino-Jarquin, 2018). Hence it has been used for several applications including detecting RNA viruses, downregulating genes by targeting RNA transcripts, imaging RNA sequences, etc. (Granados-Riveron and Aquino-Jarquin, 2018; Freije et al., 2019). RNA imaging is a powerful application because it potentially can be used as an alternative for fluorescence *in situ* hybridization (FISH) in live cells (Yang et al., 2019). Using this tool to study 3D organoids can allow for rapid real-time tracking of several genes that may not just be involved in causing diseases but also in elucidating healthy development and differentiation of stem cells.

In this review we addressed several examples in the use of CRISPR Cas9 tools to repair defective genes in diseased organoids. The ultimate goal of such studies is to aid in the development of emerging treatments that employ gene therapy, chimeric antigen receptor T cell therapy or cell therapy. This requires *in vivo* delivery of the Cas9-sgRNA complex, and examples of this have been shown for rodent disease models (Ding et al., 2014; Bakondi et al., 2016; Gao et al., 2018). While several therapeutic challenges remain, these animal models suggest that it may be possible to target defective genes in humans. Therefore, addressing these areas, particularly in patient-derived hiPSCs that are differentiated into specific adult stem cells and terminal progeny represents an opportunity to bring the “bedside” to the “bench” and advance therapeutic options for patients.

## Author Contributions

SG and AR wrote the manuscript along with JD. JD edited the manuscript. All authors contributed to the article and approved the submitted version.

## Conflict of Interest

The authors declare that the research was conducted in the absence of any commercial or financial relationships that could be construed as a potential conflict of interest.
